# Evaluation of G-Protein-Coupled Bile Acid Receptor 1 (TGR5) Levels in Intrahepatic Cholestasis of Pregnancy

**DOI:** 10.7759/cureus.19654

**Published:** 2021-11-17

**Authors:** Kader Irak, Mehmet Bayram, Sami Cifci, Zuat Acar, Cemal Kazezoglu, Deniz Ogutmen Koc, Oyku Arslan

**Affiliations:** 1 Gastroenterology, Basaksehir Cam and Sakura City Hospital, Istanbul, TUR; 2 Gastroenterology, University of Health Sciences, Kanuni Sultan Suleyman Training and Research Hospital, Istanbul, TUR; 3 Gynecology and Obstetrics, University of Health Sciences, Kanuni Sultan Suleyman Training and Research Hospital, Istanbul, TUR; 4 Biochemistry, University of Health Sciences, Kanuni Sultan Suleyman Training and Research Hospital, Istanbul, TUR; 5 Gastroenterology, University of Health Sciences, Gaziosmanpasa Training and Research Hospital, Istanbul, TUR; 6 Hematology, Basaksehir Cam and Sakura City Hospital, Istanbul, TUR

**Keywords:** bile acid, itching, intrahepatic cholestasis of pregnancy, pregnancy-related liver disease, tgr5 agonists, hepatology

## Abstract

Background and objective

Intrahepatic cholestasis of pregnancy (ICP) is the most common pregnancy-related liver disease. G-protein-coupled bile acid receptor 1 (TGR5) agonists might be beneficial in ICP treatment. In this study, we aimed to investigate the relationship of serum TGR5 levels with ICP and associated itching.

Methods

Sixty-three pregnant women diagnosed with ICP based on a serum bile acid level of >10 µmol/L (patient group) and 47 healthy pregnant women as a control group were included in the study. In the patient group, ursodeoxycholic acid (UDCA) therapy was given at a dose of 15 mg/kg from the time of diagnosis until the termination of pregnancy. Serum transaminase levels were measured at the beginning and within 15 days after the onset of treatment, and the dose was increased in patients who were unresponsive to treatment.

Results

Bile acid level was found to be between 10-39 µmol/L in 61.9% of the ICP patients, and it was ≥40 µmol/L in 38.1% of the patients. The majority of the patients responded well to the treatment with UDCA. The mean TGR5 level was significantly higher in the patient group compared to the control group (0.98 ±0.95 ng/mL vs. 0.74 ±0.23 ng/mL, p=0.032). In the patient group, TGR5 level showed negative correlations with age and red cell distribution width and a positive correlation with lactate dehydrogenase level and lymphocyte count.

Conclusions

Based on our findings, it can be suggested that TGR5 may have a role in the pathogenesis but has no impact on the prognosis of the condition.

## Introduction

Intrahepatic cholestasis of pregnancy (ICP) is the most commonly encountered pregnancy-related liver disease. It usually occurs in the third trimester of pregnancy and is characterized by typical itching and increased serum transaminase and bile acid levels [1]. Endocrine, genetic, and environmental factors play a role in the etiology of ICP [2]. Its incidence shows substantial variations among countries. In general, the incidence of ICP varies between 0.2% and 2%, but it is reported that the incidence is as high as 22% in some populations [2].

The diagnosis of ICP is made based on typical complaints of palmar and plantar itching and elevated levels of serum bile acid [3]. The most sensitive and specific diagnostic marker is a serum bile acid level of >10 µmol/L [4]. ICP poses minimal risk for the mother; its symptoms regress with birth but may recur in subsequent pregnancies [3]. However, critical adverse effects have been reported in the fetus. It has been demonstrated that the risk of fetal complications is higher in cases with a serum bile acid level of >40 µmol/L. ICP-induced fetal complications include anoxia, meconium-stained amniotic fluid, prematurity, perinatal death, fetal distress, and stillbirth [3,4]. Such serious impacts on fetuses highlight the importance of early diagnosis and treatment of ICP. In their pilot study, Tolunay et al. found a significant positive association between first-trimester aspartate aminotransferase to platelet ratio index (APRI) score and third-trimester fasting bile acid level. The APRI score in the first trimester seems to predict the development of ICP in the last trimester of pregnancy [5].

The treatment of ICP is symptom-oriented. It focuses on relieving itching since itching is very troublesome for the patients, increases at night, and disturbs sleep. Ursodeoxycholic acid (UDCA) is beneficial in relieving itching by lowering bile acid levels, but there is scant evidence that it reduces perinatal complications [6]. Therefore, more effective therapies are required. The development of new therapies, however, requires more information about the pathogenesis of the disease.

Synthesis and regulation of bile acids are provided by nuclear and membrane receptors. Among these bile acid-activated receptors, farnesoid X receptor and G-protein-coupled bile acid receptor 1 (GPBAR1; TGR5) have been comprehensively studied [7]. The activation of farnesoid X receptor and TGR5 results in the decreased burden of hepatic bile salt, improved insulin sensitivity and glucose regulation, and increased energy consumption and anti-inflammatory effect [7]. TGR5 agonists might be used as potential drugs for some metabolic disorders, inflammation, and digestive disorders. Activated TGR5 may play a role in the treatment of various metabolic disorders such as type 2 diabetes and obesity [8]. In the light of this information, it is thought that TGR5 agonists might be beneficial in the treatment of ICP as well. Furthermore, it could be possible to diagnose ICP early, to detect patients who potentially would have severe disease, and foresee the risks in subsequent pregnancies by measuring TGR5 levels, and thereby minimize the potential risks for the fetus and the mother by closely monitoring the patients. Based on this hypothesis, we aimed to investigate the relationship of serum TGR5 levels with ICP and associated itching in this study.

## Materials and methods

Patients with itching and serum transaminase levels higher than normal during pregnancy and who consulted gastroenterology and were accordingly diagnosed with ICP based on a serum bile acid level of >10 µmol/L were included in the study. Patients with pregnancy-related diseases such as gestational diabetes, preeclampsia, eclampsia, or fatty liver of pregnancy, and those with a history of cholestatic liver disease (primary biliary cholangitis, primary sclerosing cholangitis) were excluded. A control group was formed comprising women having normal pregnancies. Written informed consent was obtained from the participants. The study protocol adhered to the ethical guidelines of the 1975 Declaration of Helsinki. The study was approved by the Ethics Committee of Kanuni Sultan Suleyman Training and Research Hospital (No: KAEK/2020.07.149. Date: 23.07.2020).

The age of the participants, number of pregnancies, and gestational age were recorded for both the patient and control groups. Routine complete blood count and biochemical parameters were measured. In addition, fasting blood samples were collected in plain tubes for TGR5 measurement, and they were centrifuged at 3000 rpm for 10 minutes to obtain serum samples. TGR5 level was measured in ng/mL. In the patient group, UDCA therapy was given at a dose of 15 mg/kg from the time of diagnosis of ICP until the termination of pregnancy. Serum transaminase levels were measured again within 15 days after the onset of treatment, and the dose was increased in patients who were unresponsive to treatment.

The PASW Statistics for Windows, Version 18.0 (IBM, Armonk, NY) was used for statistical analysis. Descriptive statistics were expressed as number and percentage for categorical variables and as mean, standard deviation, median, first quartile (Q1) (percentile 25), and third quartile (Q3) (percentile 75) for numerical variables. The suitability of variables to normal distribution was analyzed using visual (histogram and probability graphics) and analytic (Kolmogorov-Smirnov/Shapiro-Wilk tests) methods. Chi-square test statistics were used for paired and multiple group comparisons of categorical variables. Paired group comparisons of numerical variables were performed using the Student’s t-test in case the condition of normal distribution was met, whereas the Mann-Whitney U test was used in case the condition of normal distribution was not met. Correlation between TGR5 level and numerical variables was analyzed by the Spearman’s rho test in case the condition of normal distribution was not met. A p-value of <0.05 was considered statistically significant.

## Results

The study comprised 63 pregnant women diagnosed with ICP as the patient group and 47 age-matched normal pregnant women as the control group. A comparison of the general characteristics between the patients and the control group is presented in Table 1. Gestational age, alanine aminotransferase, aspartate aminotransferase, total bilirubin, direct bilirubin, hemoglobin, platelet, and platelet/lymphocyte values were significantly higher and lymphocyte values were significantly lower in the ICP group when compared to the control group (Table 1).

**Table 1:** General characteristics of the patient and control groups ALT: alanine aminotransferase; AST: aspartate aminotransferase; MPV: mean platelet volume; RDW: red cell distribution width; SD: standard deviation; WBC: white blood cell

Variables	Patient group				Control group		
	N	Mean ±SD	Median (Q1-Q3)	N	Mean ±SD	Median (Q1-Q3)	P-value
Age in years	63	28.7 ±5.76	28 (24-33)	47	29.47 ±5.7	28 (25-35)	0.488
Number of pregnancies	61	2.46 ±1.46	2 (1-3)	47	2.68 ±1.27	3 (2-4)	0.252
Gestational age (weeks)	60	31.53 ±5.07	33 (31-34)	47	28.79 ±5.74	28 (23-35)	0.018
ALT, U/L	62	125.53 ±118.79	72 (48-165)	47	11 ±5.14	9 (8-13)	<0.001
AST, U/L	62	72.87 ±53.25	56.5 (38-92)	47	14.77 ±2.86	14 (13-17)	<0.001
Total bilirubin, mg/dL	60	0.57 ±0.37	0.5 (0.32-0.72)	47	0.24 ±0.09	0.23 (0.15-0.3)	<0.001
Direct bilirubin, mg/dL	60	0.38 ±0.3	0.28 (0.2-0.51)	47	0.12 ±0.03	0.11 (0.1-0.12)	<0.001
Hemoglobin, g/dL	62	11.64 ±1.33	11.65 (10.9-12.7)	47	11.15 ±1.15	11.2 (10.5-12.1)	0.049
WBC, x 10^3^/mm^3^	62	9.44 ±1.91	9.5 (8.2-10.6)	47	9.17 ±2.58	9.12 (7.92-10.85)	0.534
MPV, fL	62	11.15 ±1.13	11.15 (10.2-11.8)	47	11.06 ±1.17	11 (10.1-12)	0.609
RDW, %	62	13.51 ±1.58	13.1 (12.6-13.8)	47	13.29 ±0.96	13.2 (13-13.7)	0.518
Neutrophil, x 10^3^/mm^3^	62	6.89 ±1.8	7 (5.3-7.7)	47	7.46 ±1.65	7.71 (6.32-8.4)	0.097
Lymphocyte, x 10^3^/mm^3^	62	1.8 ±0.46	1.8 (1.6-2)	47	1.97 ±0.37	1.9 (1.6-2.3)	0.049
Platelet, x 10^3^/mm^3^	62	244.32 ±75.02	231.5 (194-281)	47	213.85 ±47.38	213 (179-238)	0.016
Neutrophil/lymphocyte	62	4.15 ±1.87	3.85 (2.85-4.7)	47	3.9 ±1.01	3.83 (3.16-4.56)	0.998
Platelet/lymphocyte	62	143.55 ±55.22	124.41 (107.33-176.43)	47	112.46 ±31.19	116.8 (91.58-131.3)	0.009

While bile acid level was between 10 µmol/L and 39 µmol/L in 61.9% of ICP patients, it was ≥40 µmol/L in 38.1% of the patients. The majority of the patients responded well to the treatment with UDCA. The median gestational age was 37 weeks and more than half of the patients (61.5%) gave birth by cesarean section. Admission to the ICU was required for 13 patients. Some characteristics of the patient group are given in Table 2.

**Table 2:** Characteristics of the patient group The values are given as numbers (%) or mean ±standard deviation and median (1st quartile-3rd quartile) GGT: gamma-glutamyl transferase; LDH: lactate dehydrogenase; UDCA: ursodeoxycholic acid

Characteristics	N	N (%)	Mean ±SD	Median (Q1-Q3)
Bile acid, µmol/L	63		40.62 ±37	27 (16-49)
Bile acid	63			
10-39 µmol/L		39 (61.9)		
≥40 µmol/L		24 (38.1)		
ALP, U/L	54		201.54 ±73.9	201.5 (144-256)
GGT, U/L	56		26.8 ±35.78	17 (11.5-29)
LDH, U/L	48		230.15 ±49.56	232.5 (197.5-263)
Itching	63			
Mild-moderate				22 (34.9)
Manifest-severe				41 (65.1)
UDCA dosage (x 250 mg cap/day)	63		3.57 ±0.73	4 (3-4)
Alleviated itching with UDCA therapy	52			41 (78.8)
ALT level with UDCA therapy	62		65.34 ±67.77	44.5 (28-69)
ALT level decreased by 50% or became normal with UDCA therapy	42			31 (73.8)
AST level with UDCA therapy	59		40.03 ±28.67	34 (22-54)
Gestational age (weeks)	52		36.75 ±1.76	37 (36-38)
Route of delivery	52			
Cesarean section				32 (61.5)
Vaginal				20 (38.5)
Admission to the intensive care unit	52			
No				39 (75)
Yes				13 (25)
Infant birth weight, g	52		2718.63 ±787.99	2862.5 (2370-3200)

**Figure 1:**
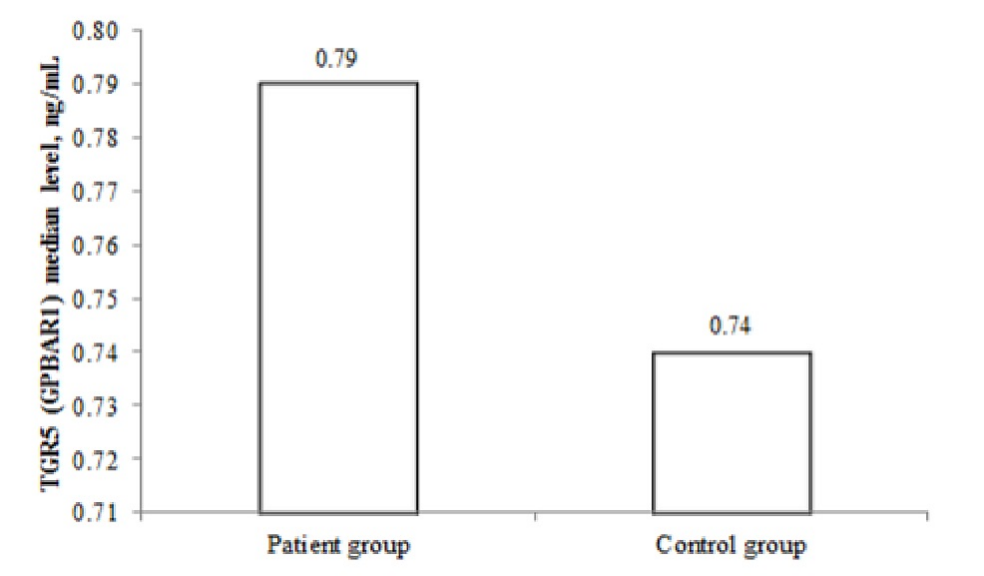
TGR5 levels in the patient and control groups

The mean ±SD and median (Q1-Q3) levels of TGR5 (GPBAR1) was 0.98 ±0.95 ng/mL and 0.79 (0.70-0.97) ng/mL, respectively, in the patient group, and 0.74 ±0.23 ng/mL and 0.74 (0.54-0.87) ng/mL, respectively, in the control group. TGR5 level was significantly higher in the patient group compared to that in the control group (p=0.032) (Figure 1).

TGR5 levels were analyzed according to the characteristics of the patients. There was no significant difference in TGR5 levels between the groups established according to the characteristics (Table 3).

**Table 3:** TGR5 levels according to the characteristics of the patients ALT: alanine aminotransferase; SD: standard deviation; Q1: first quartile; Q3: third quartile; UDCA: ursodeoxycholic acid

Variables	N	TGR5 (GPBAR1) level, ng/mL		P-value
		Mean ±SD	Median (Q1-Q3)	
Bile acid				
10-39 µmol/L	39	0.85 ±0.36	0.80 (0.30-0.95)	0.388
≥40 µmol/L	24	1.20 ±1.47	0.79 (0.70-0.97)	
Itching				
Mild-moderate	22	0.86 ±0.38	0.78 (0.70-0.86)	0.345
Manifest-severe	41	1.05 ±1.15	0.83 (0.72-1.00)	
Alleviated itching with UDCA therapy				
No	11	0.81 ±0.23	0.79 (0.72-0.95)	0.662
Yes	41	0.93 ±0.44	0.79 (0.70-1.00)	
ALT level decreased by 50% or became normal with UDCA therapy				
No	11	0.80 ±0.22	0.73 (0.62-1.00)	0.637
Yes	31	0.90 ±0.49	0.77 (0.70-0.96)	
Route of delivery				
Cesarean section	32	0.93 ±0.33	0.83 (0.73-1.06)	0.441
Vaginal	20	1.22 ±1.63	0.78 (0.67-0.94)	
Admission to the intensive care unit				
No	39	0.95 ±0.46	0.81 (0.73-1.06)	0.404
Yes	13	1.32 ±1.95	0.76 (0.63-0.95)	

In the patient group, TGR5 level showed negative correlations with age and red cell distribution width, whereas there was a positive correlation with lactate dehydrogenase level and lymphocyte count (Table 4).

**Table 4:** Correlation analysis between TGR5 (GPBAR1) and other parameters in the patient group ALP: alkaline phosphatase; ALT: alanine aminotransferase; AST: aspartate aminotransferase; GGT: gamma-glutamyl transferase; GPBAR1: G-protein-coupled bile acid receptor-1; HGB: hemoglobin; LDH: lactate dehydrogenase; MPV: mean platelet volume; RDW: red cell distribution width; UDCA: ursodeoxycholic acid; WBC: white blood cell

Variables	TGR5 (GPBAR1)		
	N	rho	P-value
Age	63	-0.457	<0.001
Gestational age	60	0.123	0.348
Number of pregnancies	61	-0.229	0.076
Bile acid	63	0.044	0.734
ALT	62	-0.047	0.715
AST	62	-0.060	0.642
Total bilirubin	60	-0.011	0.936
Direct bilirubin	60	0.097	0.459
ALP	54	0.056	0.686
GGT	56	-0.199	0.142
LDH	48	0.381	0.008
HGB	62	0.151	0.241
WBC	62	0.099	0.444
MPV	62	-0.125	0.334
RDW	62	-0.263	0.039
Neutrophil	62	0.011	0.934
Lymphocyte	62	0.325	0.010
Platelet	62	-0.015	0.906
Neutrophil/lymphocyte	62	-0.142	0.272
Platelet/lymphocyte	62	-0.235	0.066
UDCA dose	63	0.003	0.983
ALT with UDCA therapy	62	-0.076	0.558
AST with UDCA therapy	59	-0.024	0.856
Gestational age at birth	52	-0.014	0.923
Birth weight	52	0.093	0.513

## Discussion

The increasing availability of data in the last few decades about the role of bile acids as signal molecules in the regulation of metabolism has drawn the attention of researchers to this subject. There is still limited knowledge about the potential changes in the physiology of bile acids during normal pregnancy. Nevertheless, it is clearly seen in the event of ICP that pathologically elevated bile acid concentrations have a harmful impact on the mother, placenta, and developing fetus. Moreover, it has been propounded that the infants given birth by mothers with ICP become more susceptible to metabolic disorders in their future lives [9]. ICP, which is a benign condition for the mother as the symptoms relieve after birth, is associated with significant risks for the fetus [10]. Therefore, early diagnosis and treatment of ICP are especially critical for the fetus. In addition, new therapeutic options have to be devised, and a better understanding of the ICP pathogenesis would serve as a guide for the investigation of new therapies. TGR5, which is the subject of the present study, is among the molecules investigated for this purpose.

Plasma membrane receptor, TGR5, is found in many hepatic cells including sinusoidal endothelial cells, Kupffer cells, hepatic stellate cells, and small and large cholangiocytes. By means of TGR5, bile salts mediate choleretic, cell-protective, and proliferative effects. Impairments in these signal mechanisms may contribute to the development of biliary diseases [11,12]. Owing to its role in the pathogenesis of various diseases such as nonalcoholic steatohepatitis, hepatic diseases associated with cholestasis (primary sclerosing cholangitis and primary biliary cholangitis), polycystic liver disease, cholangiocarcinoma, portal hypertension, cirrhosis, and sepsis, TGR5 is being investigated as the potential therapeutic target in these diseases [13-16]. The activation of TGR5 has been reported to be promising in the treatment of obesity, atherosclerosis, and non-alcoholic fatty liver disease [17]. However, although data from preclinical studies about TGR5-targeted therapies are promising, it has been reported that clinical studies are inadequate [18]. TGR5 is thought to play a role in the pathogenesis of ICP, especially in relation to itching [19]. In the present study, TGR5 levels were significantly higher in pregnant women with ICP as compared to those of the normal pregnant women (median: 0.79 ng/mL vs. 0.74 ng/mL, p=0.032), indicating that TGR5 might be playing a role in the pathogenesis of cholestasis.

The main goal of the treatment of ICP is to eliminate itching. UDCA, which is recommended as the first-line treatment in some guidelines, is the most commonly used agent and has been shown to improve itching and laboratory abnormalities [20]. In their meta-analysis, Kong et al. [21] found UDCA to be effective and safe in the treatment of ICP. Nevertheless, some women are unresponsive to UDCA therapy [22]. In the present study, treatment with UDCA alleviated itching in 78.8% and decreased alanine aminotransferase levels by 50% or to normal ranges in 73.8% of the patients.

Itching is a common symptom in cholestatic liver diseases. Treatment of this troublesome symptom that impairs the quality of life is challenging [23,24]. The underlying mechanism of itching remains unclear. The role of various factors including bile acids, opioids, steroids, and lysophosphatidic acid has been discussed, but none of them was considered as the key mediator [23]. It has been reported that elevated bile acid concentration in circulation and in the tissues during cholestasis causes itching by activating TGR5 [24-26]. In the present study, TGR5 was higher in patients with manifest-severe itching as compared to those with mild-moderate itching even though the difference did not reach statistical significance (median: 0.83 ng/mL vs. 0.78 ng/mL, p=0.345). Nevertheless, no difference was found between the TGR5 levels of the patients showing improvement in itching with UDC therapy in comparison to those without improvement (median: 0.79 ng/mL vs. 0.79 ng/mL, p=0.662).

It has been reported that mothers with ICP may be at risk of unfavorable conditions such as liver diseases, metabolic disorders, and cancer in the long term following birth [27]. Therefore, long-term follow-up of the mothers after birth is critical for detecting subsequent impacts of ICP.

It is known that severe cholestasis is associated with neonatal morbidity [28]. In the present study, bile acid concentration was ≥40 µmol/L in 38.1% of ICP patients. No significant difference was found between those with a bile acid concentration of ≥40 µmol/L and those with a bile acid concentration between 10 and 39 µmol/L in terms of TGR5 levels (median: 0.79 ng/mL vs. 0.80 ng/mL, p=0.388).

Birth at 36th gestational week in women with ICP has been reported to be the optimum birth strategy, minimizing the risk of fetal complications [29,30]. In the present study, the mean gestational age at birth in ICP patients was 36.75 ±1.76 weeks (median: 37 weeks). Of the patients, 61.5% gave birth by cesarean section, and admission to the ICU was required for 25%. The route of delivery or admission to the ICU was not found to be associated with TGR5 levels. Correlation analysis also showed no significant correlation between TGR5 levels and concentrations of bile acids and transaminases, which are the indicators of disease severity.

The major limitations of the study are that the data were collected retrospectively and the study was conducted at a single center. In addition, the direction of the relationship between TGR5 and ICP in pregnant women could not be clearly demonstrated in the study, and a cause-effect relationship could not be shown due to the study design.

## Conclusions

The present study found significantly higher TGR5 levels in ICP patients as compared to normal pregnant women. Nevertheless, no relationship was found between TGR5 levels and disease severity (bile acid and transaminase concentrations and severity of itching), response to treatment (decrease in itching and alanine aminotransferase levels), route of delivery, or admission to the ICU. This topic requires further large-scale studies with long-term follow-ups that include the postpartum period as well.
